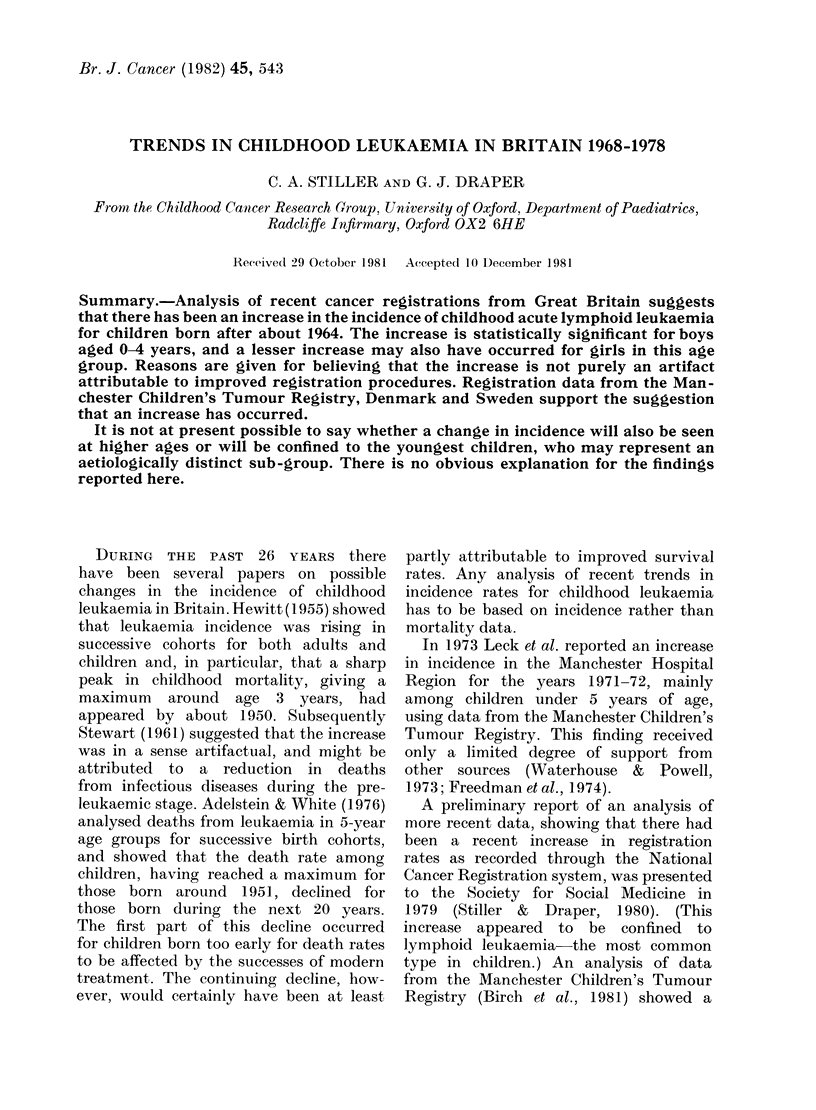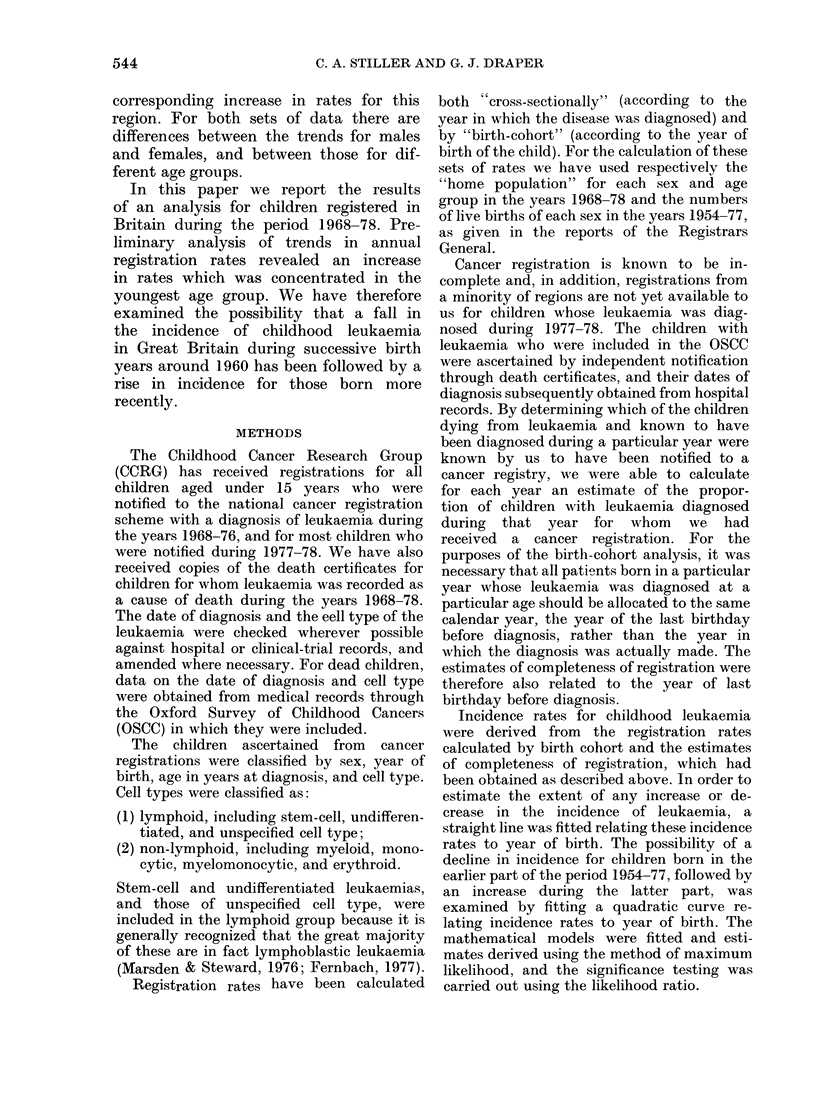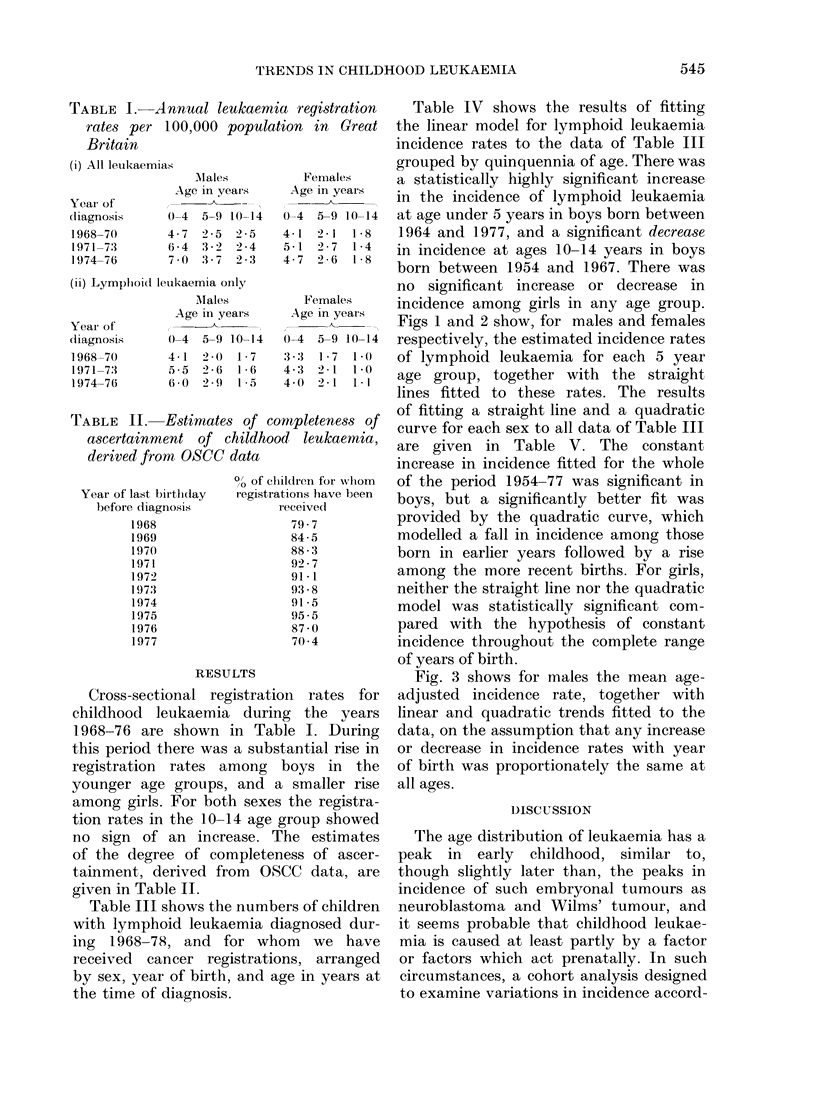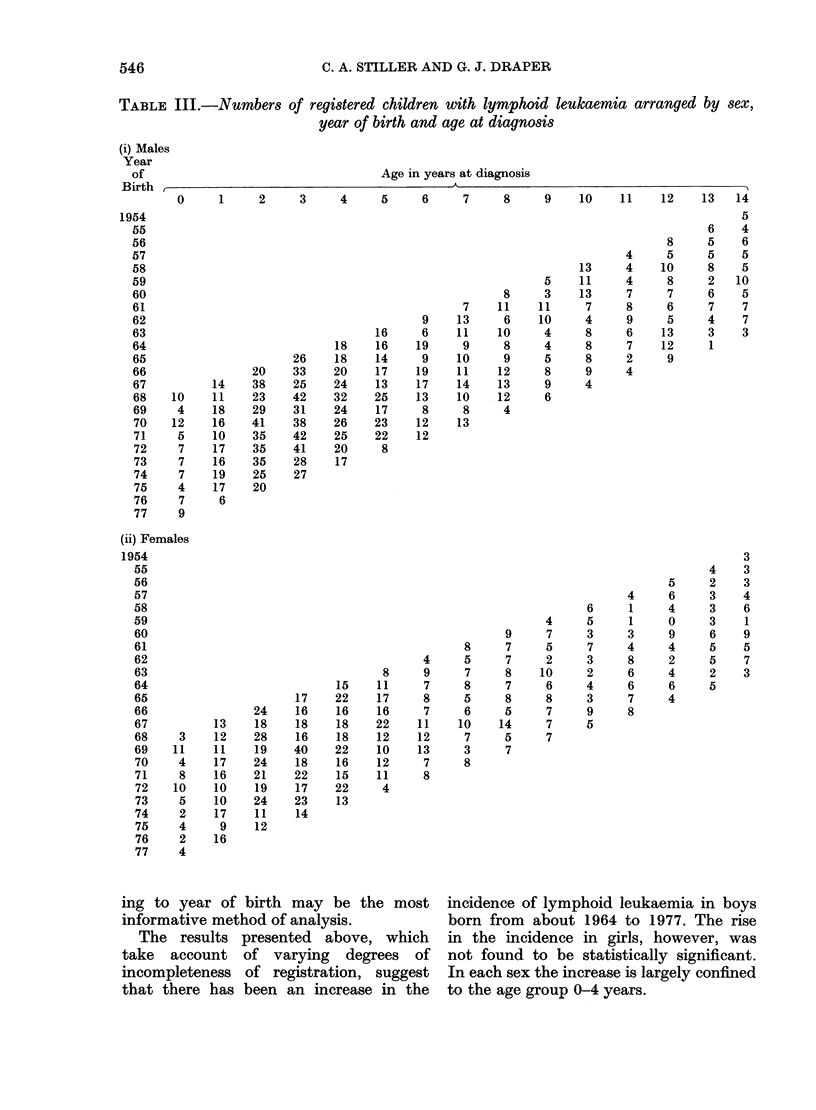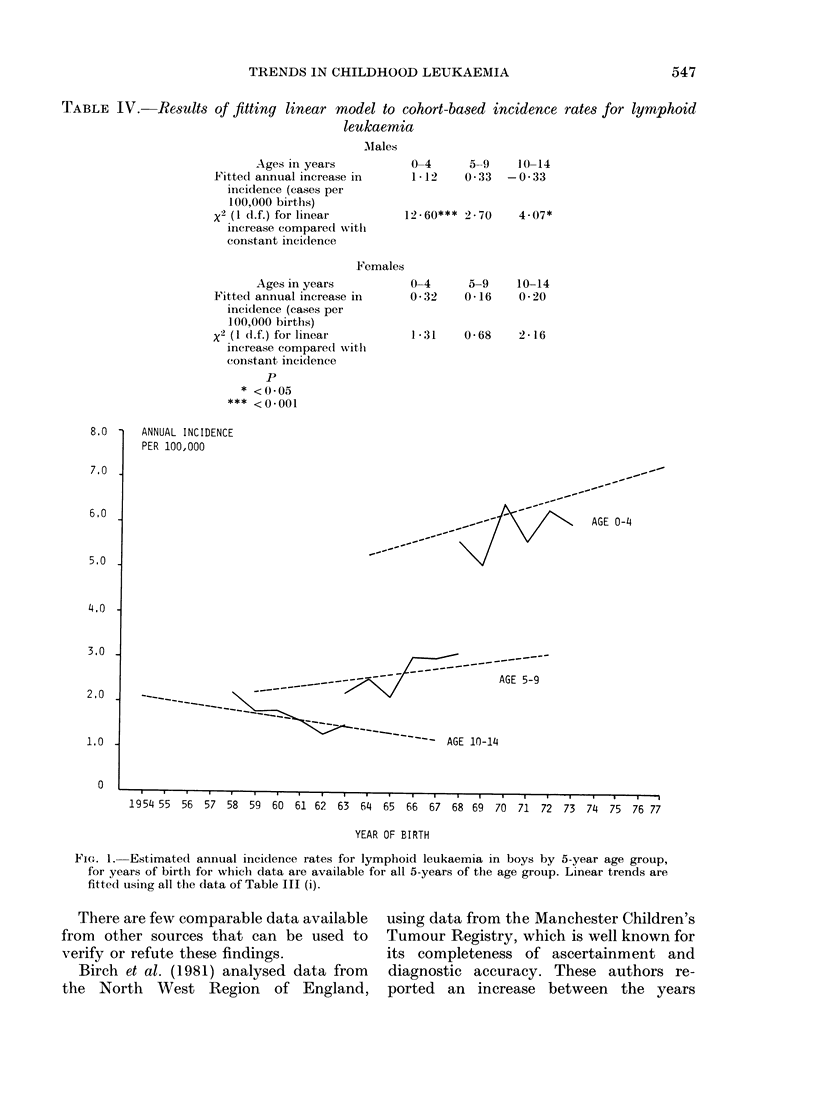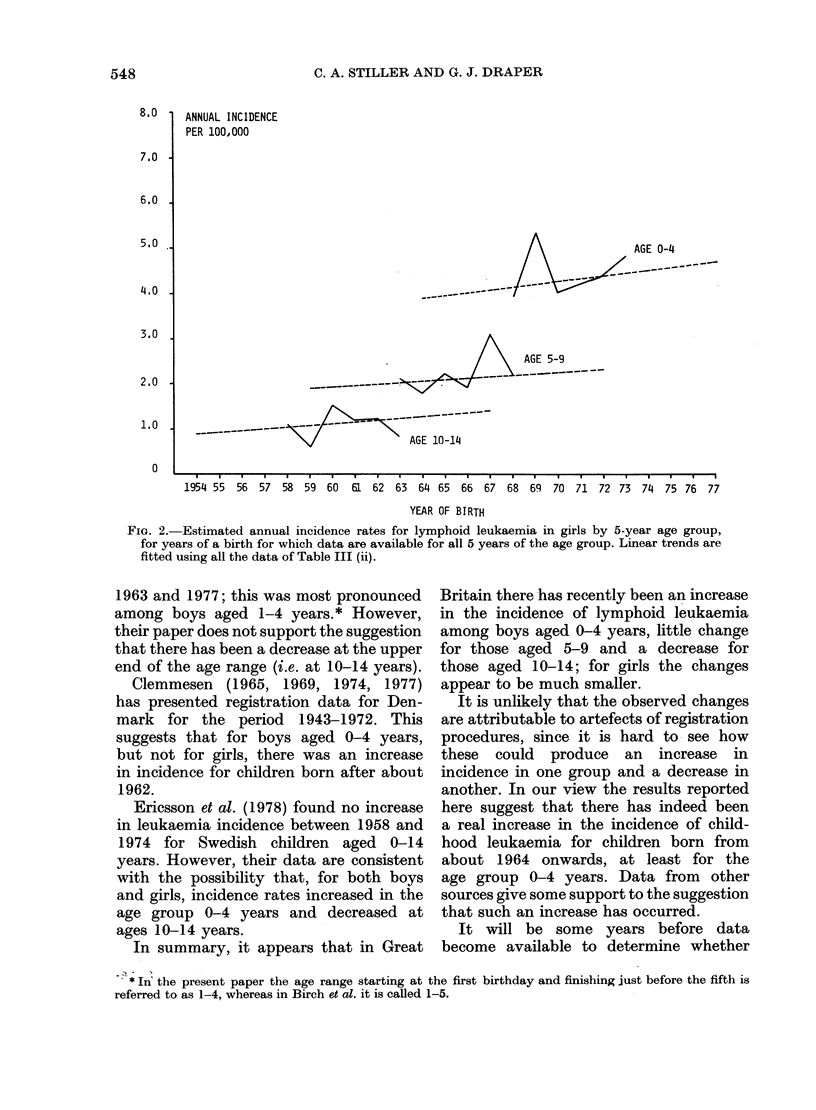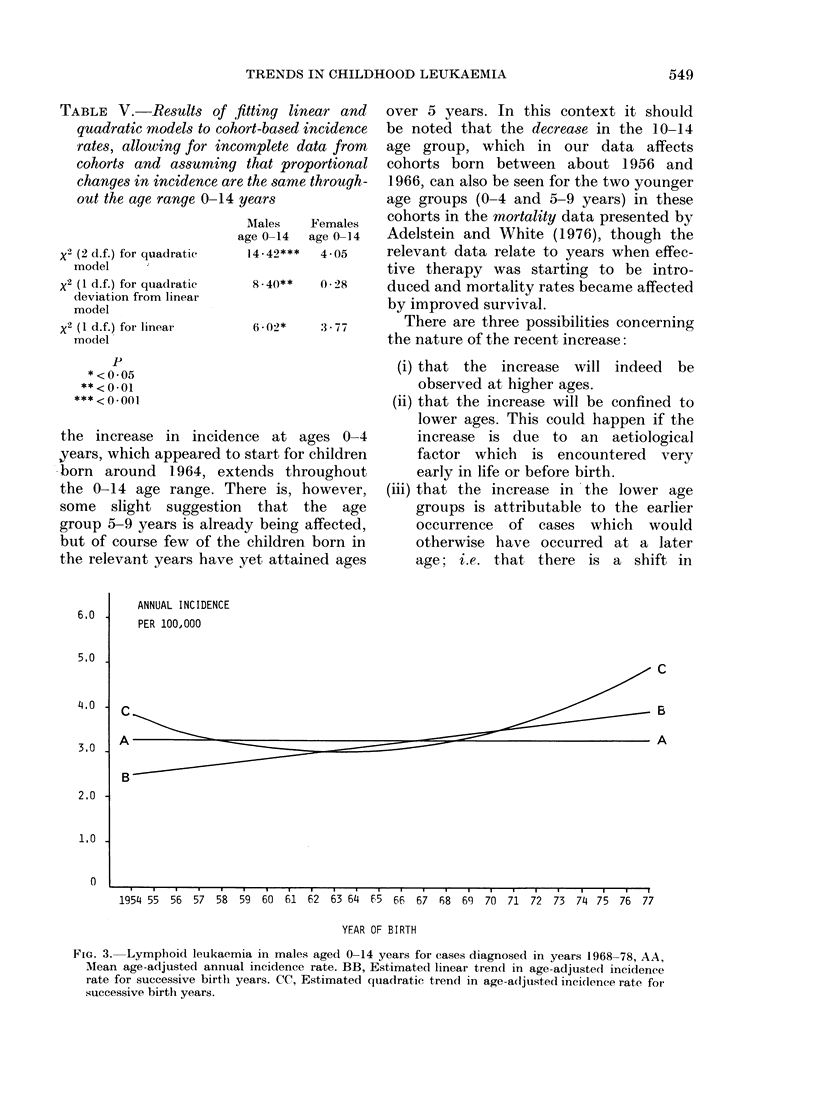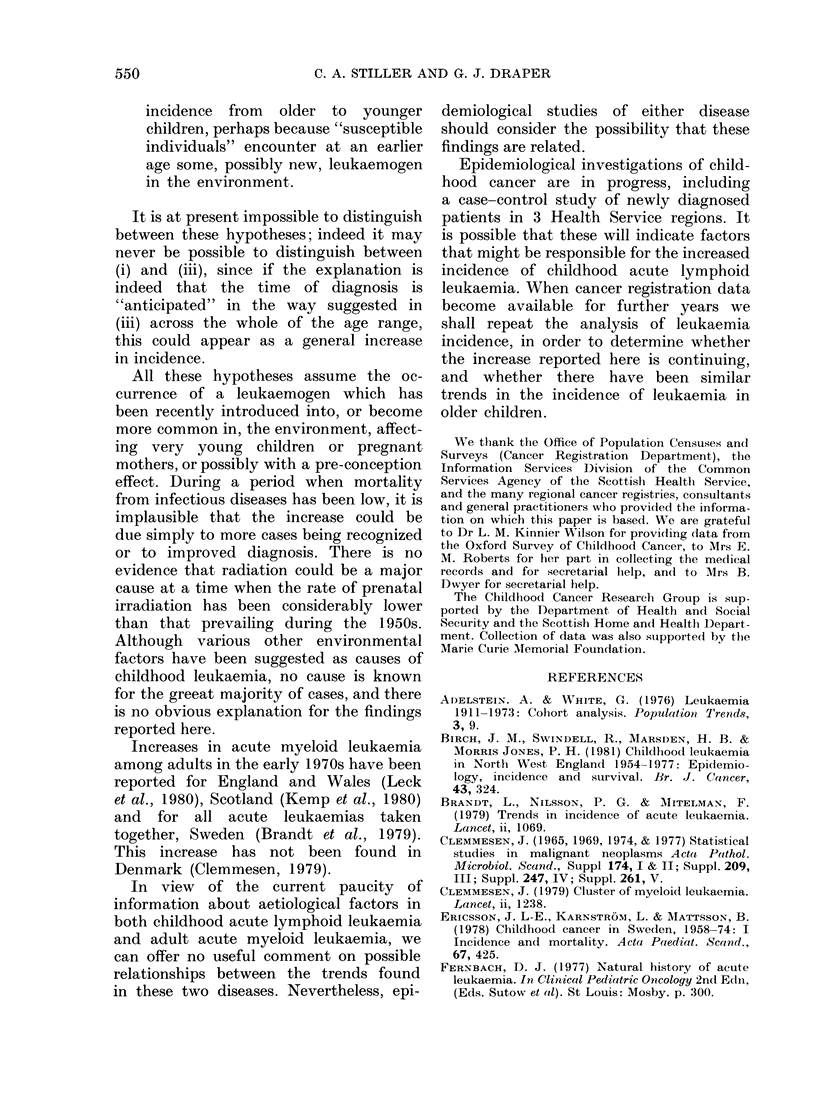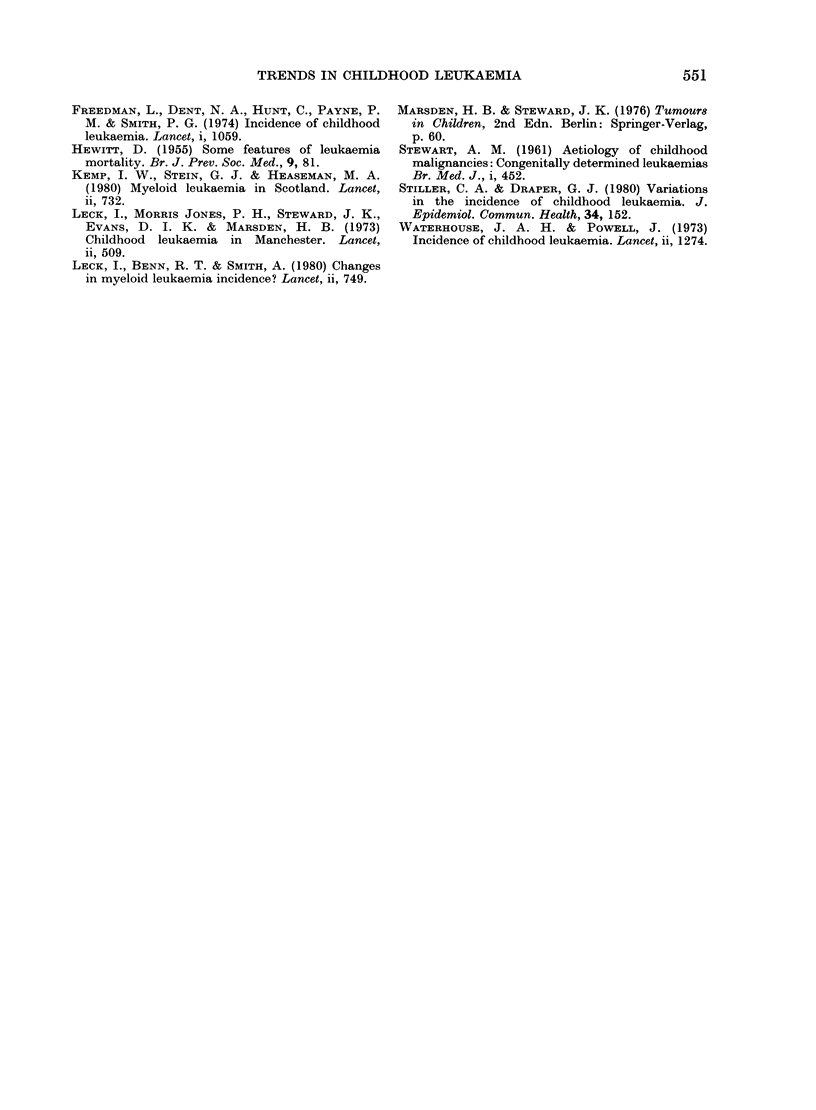# Trends in childhood leukaemia in Britain 1968-1978.

**DOI:** 10.1038/bjc.1982.90

**Published:** 1982-04

**Authors:** C. A. Stiller, G. J. Draper

## Abstract

Analysis of recent cancer registrations from Great Britain suggests that there has been an increase in the incidence of childhood acute lymphoid leukaemia for children born after about 1964. The increase is statistically significant for boys aged 0-4 years, and a lesser increase may also have occurred for girls in this age group. Reasons are given for believing that the increase is not purely an artifact attributable to improved registration procedures. Registration data from the Manchester Children's Tumour Registry, Denmark and Sweden support the suggestion that an increase h as occurred. It is not at present possible to say whether a change in incidence will also be seen at higher ages or will be confined to the youngest children, who may represent an aetiologically distinct sub-group. There is no obvious explanation for the findings reported here.


*Br. J. Cancer* (1982) **45**, 543

### TRENDS IN CHILDHOOD LEUKAEMIA IN BRITAIN 1968-1978

C. A. STILLER AND G. J. DRAPER

*From the Childhood Cancer Research Group, University of Oxford, Department of Paediatrics, Radcliffe Infirmary, Oxford OX2 6HE*

Received 29 October 1981 Accepted 10 December 1981

**Summary.—Analysis of recent cancer registrations from Great Britain suggests that there has been an increase in the incidence of childhood acute lymphoid leukaemia for children born after about 1964. The increase is statistically significant for boys aged 0–4 years, and a lesser increase may also have occurred for girls in this age group. Reasons are given for believing that the increase is not purely an artifact attributable to improved registration procedures. Registration data from the Manchester Children's Tumour Registry, Denmark and Sweden support the suggestion that an increase has occurred.**

**It is not at present possible to say whether a change in incidence will also be seen at higher ages or will be confined to the youngest children, who may represent an aetiologically distinct sub-group. There is no obvious explanation for the findings reported here.**

DURING THE PAST 26 YEARS there have been several papers on possible changes in the incidence of childhood leukaemia in Britain. Hewitt (1955) showed that leukaemia incidence was rising in successive cohorts for both adults and children and, in particular, that a sharp peak in childhood mortality, giving a maximum around age 3 years, had appeared by about 1950. Subsequently Stewart (1961) suggested that the increase was in a sense artifactual, and might be attributed to a reduction in deaths from infectious diseases during the pre-leukaemic stage. Adelstein & White (1976) analysed deaths from leukaemia in 5-year age groups for successive birth cohorts, and showed that the death rate among children, having reached a maximum for those born around 1951, declined for those born during the next 20 years. The first part of this decline occurred for children born too early for death rates to be affected by the successes of modern treatment. The continuing decline, however, would certainly have been at least partly attributable to improved survival rates. Any analysis of recent trends in incidence rates for childhood leukaemia has to be based on incidence rather than mortality data.

In 1973 Leck *et al.* reported an increase in incidence in the Manchester Hospital Region for the years 1971–72, mainly among children under 5 years of age, using data from the Manchester Children's Tumour Registry. This finding received only a limited degree of support from other sources (Waterhouse & Powell, 1973; Freedman *et al.*, 1974).

A preliminary report of an analysis of more recent data, showing that there had been a recent increase in registration rates as recorded through the National Cancer Registration system, was presented to the Society for Social Medicine in 1979 (Stiller & Draper, 1980). (This increase appeared to be confined to lymphoid leukaemia—the most common type in children.) An analysis of data from the Manchester Children's Tumour Registry (Birch *et al.*, 1981) showed a corresponding increase in rates for this region. For both sets of data there are differences between the trends for males and females, and between those for different age groups.

In this paper we report the results of an analysis for children registered in Britain during the period 1968–78. Preliminary analysis of trends in annual registration rates revealed an increase in rates which was concentrated in the youngest age group. We have therefore examined the possibility that a fall in the incidence of childhood leukaemia in Great Britain during successive birth years around 1960 has been followed by a rise in incidence for those born more recently.

### METHODS

The Childhood Cancer Research Group (CCRG) has received registrations for all children aged under 15 years who were notified to the national cancer registration scheme with a diagnosis of leukaemia during the years 1968–76, and for most children who were notified during 1977–78. We have also received copies of the death certificates for children for whom leukaemia was recorded as a cause of death during the years 1968–78. The date of diagnosis and the eell type of the leukaemia were checked wherever possible against hospital or clinical-trial records, and amended where necessary. For dead children, data on the date of diagnosis and cell type were obtained from medical records through the Oxford Survey of Childhood Cancers (OSCC) in which they were included.

The children ascertained from cancer registrations were classified by sex, year of birth, age in years at diagnosis, and cell type. Cell types were classified as:

(1) lymphoid, including stem-cell, undifferentiated, and unspecified cell type;
(2) non-lymphoid, including myeloid, monocytic, myelomonocytic, and erythroid.

Stem-cell and undifferentiated leukaemias, and those of unspecified cell type, were included in the lymphoid group because it is generally recognized that the great majority of these are in fact lymphoblastic leukaemia (Marsden & Steward, 1976; Fernbach, 1977).

Registration rates have been calculated both "cross-sectionally" (according to the year in which the disease was diagnosed) and by "birth-cohort" (according to the year of birth of the child). For the calculation of these sets of rates we have used respectively the "home population" for each sex and age group in the years 1968–78 and the numbers of live births of each sex in the years 1954–77, as given in the reports of the Registrars General.

Cancer registration is known to be incomplete and, in addition, registrations from a minority of regions are not yet available to us for children whose leukaemia was diagnosed during 1977–78. The children with leukaemia who were included in the OSCC were ascertained by independent notification through death certificates, and their dates of diagnosis subsequently obtained from hospital records. By determining which of the children dying from leukaemia and known to have been diagnosed during a particular year were known by us to have been notified to a cancer registry, we were able to calculate for each year an estimate of the proportion of children with leukaemia diagnosed during that year for whom we had received a cancer registration. For the purposes of the birth-cohort analysis, it was necessary that all patients born in a particular year whose leukaemia was diagnosed at a particular age should be allocated to the same calendar year, the year of the last birthday before diagnosis, rather than the year in which the diagnosis was actually made. The estimates of completeness of registration were therefore also related to the year of last birthday before diagnosis.

Incidence rates for childhood leukaemia were derived from the registration rates calculated by birth cohort and the estimates of completeness of registration, which had been obtained as described above. In order to estimate the extent of any increase or decrease in the incidence of leukaemia, a straight line was fitted relating these incidence rates to year of birth. The possibility of a decline in incidence for children born in the earlier part of the period 1954–77, followed by an increase during the latter part, was examined by fitting a quadratic curve relating incidence rates to year of birth. The mathematical models were fitted and estimates derived using the method of maximum likelihood, and the significance testing was carried out using the likelihood ratio.

TABLE I.—*Annual leukaemia registration rates per* 100,000 *population in Great Britain*

(i) All leukaemias

| Year of diagnosis | Males Age in years 0–4 | 5–9 | 10–14 | Females Age in years 0–4 | 5–9 | 10–14 |
|---|---|---|---|---|---|---|
| 1968–70 | 4·7 | 2·5 | 2·5 | 4·1 | 2·1 | 1·8 |
| 1971–73 | 6·4 | 3·2 | 2·4 | 5·1 | 2·7 | 1·4 |
| 1974–76 | 7·0 | 3·7 | 2·3 | 4·7 | 2·6 | 1·8 |

(ii) Lymphoid leukaemia only

| Year of diagnosis | Males Age in years 0–4 | 5–9 | 10–14 | Females Age in years 0–4 | 5–9 | 10–14 |
|---|---|---|---|---|---|---|
| 1968–70 | 4·1 | 2·0 | 1·7 | 3·3 | 1·7 | 1·0 |
| 1971–73 | 5·5 | 2·6 | 1·6 | 4·3 | 2·1 | 1·0 |
| 1974–76 | 6·0 | 2·9 | 1·5 | 4·0 | 2·1 | 1·1 |

TABLE II.—*Estimates of completeness of ascertainment of childhood leukaemia, derived from OSCC data*

| Year of last birthday before diagnosis | % of children for whom registrations have been received |
|---|---|
| 1968 | 79·7 |
| 1969 | 84·5 |
| 1970 | 88·3 |
| 1971 | 92·7 |
| 1972 | 91·1 |
| 1973 | 93·8 |
| 1974 | 91·5 |
| 1975 | 95·5 |
| 1976 | 87·0 |
| 1977 | 70·4 |

### RESULTS

Cross-sectional registration rates for childhood leukaemia during the years 1968–76 are shown in Table I. During this period there was a substantial rise in registration rates among boys in the younger age groups, and a smaller rise among girls. For both sexes the registration rates in the 10–14 age group showed no sign of an increase. The estimates of the degree of completeness of ascertainment, derived from OSCC data, are given in Table II.

Table III shows the numbers of children with lymphoid leukaemia diagnosed during 1968–78, and for whom we have received cancer registrations, arranged by sex, year of birth, and age in years at the time of diagnosis.

Table IV shows the results of fitting the linear model for lymphoid leukaemia incidence rates to the data of Table III grouped by quinquennia of age. There was a statistically highly significant increase in the incidence of lymphoid leukaemia at age under 5 years in boys born between 1964 and 1977, and a significant *decrease* in incidence at ages 10–14 years in boys born between 1954 and 1967. There was no significant increase or decrease in incidence among girls in any age group. Figs 1 and 2 show, for males and females respectively, the estimated incidence rates of lymphoid leukaemia for each 5 year age group, together with the straight lines fitted to these rates. The results of fitting a straight line and a quadratic curve for each sex to all data of Table III are given in Table V. The constant increase in incidence fitted for the whole of the period 1954–77 was significant in boys, but a significantly better fit was provided by the quadratic curve, which modelled a fall in incidence among those born in earlier years followed by a rise among the more recent births. For girls, neither the straight line nor the quadratic model was statistically significant compared with the hypothesis of constant incidence throughout the complete range of years of birth.

Fig. 3 shows for males the mean age-adjusted incidence rate, together with linear and quadratic trends fitted to the data, on the assumption that any increase or decrease in incidence rates with year of birth was proportionately the same at all ages.

### DISCUSSION

The age distribution of leukaemia has a peak in early childhood, similar to, though slightly later than, the peaks in incidence of such embryonal tumours as neuroblastoma and Wilms' tumour, and it seems probable that childhood leukaemia is caused at least partly by a factor or factors which act prenatally. In such circumstances, a cohort analysis designed to examine variations in incidence accord-

TABLE III.—*Numbers of registered children with lymphoid leukaemia arranged by sex, year of birth and age at diagnosis*

(i) Males

| Year of Birth | Age in years at diagnosis | | | | | | | | | | | | | | |
|---|---|---|---|---|---|---|---|---|---|---|---|---|---|---|---|
| | 0 | 1 | 2 | 3 | 4 | 5 | 6 | 7 | 8 | 9 | 10 | 11 | 12 | 13 | 14 |
| 1954 | | | | | | | | | | | | | | | 5 |
| 55 | | | | | | | | | | | | | | 6 | 4 |
| 56 | | | | | | | | | | | | | 8 | 5 | 6 |
| 57 | | | | | | | | | | | | 4 | 5 | 5 | 5 |
| 58 | | | | | | | | | | | 13 | 4 | 10 | 8 | 5 |
| 59 | | | | | | | | | | 5 | 11 | 4 | 8 | 2 | 10 |
| 60 | | | | | | | | | 8 | 3 | 13 | 7 | 7 | 6 | 5 |
| 61 | | | | | | | | 7 | 11 | 11 | 7 | 8 | 6 | 7 | 7 |
| 62 | | | | | | | 9 | 13 | 6 | 10 | 4 | 9 | 5 | 4 | 7 |
| 63 | | | | | | 16 | 6 | 11 | 10 | 4 | 8 | 6 | 13 | 3 | 3 |
| 64 | | | | | 18 | 16 | 19 | 9 | 8 | 4 | 8 | 7 | 12 | 1 | |
| 65 | | | | 26 | 18 | 14 | 9 | 10 | 9 | 5 | 8 | 2 | 9 | | |
| 66 | | | 20 | 33 | 20 | 17 | 19 | 11 | 12 | 8 | 9 | 4 | | | |
| 67 | | 14 | 38 | 25 | 24 | 13 | 17 | 14 | 13 | 9 | 4 | | | | |
| 68 | 10 | 11 | 23 | 42 | 32 | 25 | 13 | 10 | 12 | 6 | | | | | |
| 69 | 4 | 18 | 29 | 31 | 24 | 17 | 8 | 8 | 4 | | | | | | |
| 70 | 12 | 16 | 41 | 38 | 26 | 23 | 12 | 13 | | | | | | | |
| 71 | 5 | 10 | 35 | 42 | 25 | 22 | 12 | | | | | | | | |
| 72 | 7 | 17 | 35 | 41 | 20 | 8 | | | | | | | | | |
| 73 | 7 | 16 | 35 | 28 | 17 | | | | | | | | | | |
| 74 | 7 | 19 | 25 | 27 | | | | | | | | | | | |
| 75 | 4 | 17 | 20 | | | | | | | | | | | | |
| 76 | 7 | 6 | | | | | | | | | | | | | |
| 77 | 9 | | | | | | | | | | | | | | |

(ii) Females

| Year of Birth | 0 | 1 | 2 | 3 | 4 | 5 | 6 | 7 | 8 | 9 | 10 | 11 | 12 | 13 | 14 |
|---|---|---|---|---|---|---|---|---|---|---|---|---|---|---|---|
| 1954 | | | | | | | | | | | | | | | 3 |
| 55 | | | | | | | | | | | | | | 4 | 3 |
| 56 | | | | | | | | | | | | | 5 | 2 | 3 |
| 57 | | | | | | | | | | | | 4 | 6 | 3 | 4 |
| 58 | | | | | | | | | | | 6 | 1 | 4 | 3 | 6 |
| 59 | | | | | | | | | | 4 | 5 | 1 | 0 | 3 | 1 |
| 60 | | | | | | | | | 9 | 7 | 3 | 3 | 9 | 6 | 9 |
| 61 | | | | | | | | 8 | 7 | 5 | 7 | 4 | 4 | 5 | 5 |
| 62 | | | | | | | 4 | 5 | 7 | 2 | 3 | 8 | 2 | 5 | 7 |
| 63 | | | | | | 8 | 9 | 7 | 8 | 10 | 2 | 6 | 4 | 2 | 3 |
| 64 | | | | | 15 | 11 | 7 | 8 | 7 | 6 | 4 | 6 | 6 | 5 | |
| 65 | | | | 17 | 22 | 17 | 8 | 5 | 8 | 8 | 3 | 7 | 4 | | |
| 66 | | | 24 | 16 | 16 | 16 | 7 | 6 | 5 | 7 | 9 | 8 | | | |
| 67 | | 13 | 18 | 18 | 18 | 22 | 11 | 10 | 14 | 7 | 5 | | | | |
| 68 | 3 | 12 | 28 | 16 | 18 | 12 | 12 | 7 | 5 | 7 | | | | | |
| 69 | 11 | 11 | 19 | 40 | 22 | 10 | 13 | 3 | 7 | | | | | | |
| 70 | 4 | 17 | 24 | 18 | 16 | 12 | 7 | 8 | | | | | | | |
| 71 | 8 | 16 | 21 | 22 | 15 | 11 | 8 | | | | | | | | |
| 72 | 10 | 10 | 19 | 17 | 22 | 4 | | | | | | | | | |
| 73 | 5 | 10 | 24 | 23 | 13 | | | | | | | | | | |
| 74 | 2 | 17 | 11 | 14 | | | | | | | | | | | |
| 75 | 4 | 9 | 12 | | | | | | | | | | | | |
| 76 | 2 | 16 | | | | | | | | | | | | | |
| 77 | 4 | | | | | | | | | | | | | | |

ing to year of birth may be the most informative method of analysis.

The results presented above, which take account of varying degrees of incompleteness of registration, suggest that there has been an increase in the incidence of lymphoid leukaemia in boys born from about 1964 to 1977. The rise in the incidence in girls, however, was not found to be statistically significant. In each sex the increase is largely confined to the age group 0–4 years.

TABLE IV.—*Results of fitting linear model to cohort-based incidence rates for lymphoid leukaemia*

| Males | | | |
|---|---|---|---|
| Ages in years | 0–4 | 5–9 | 10–14 |
| Fitted annual increase in incidence (cases per 100,000 births) | 1·12 | 0·33 | −0·33 |
| $\chi^2$ (1 d.f.) for linear increase compared with constant incidence | 12·60*** | 2·70 | 4·07* |

| Females | | | |
|---|---|---|---|
| Ages in years | 0–4 | 5–9 | 10–14 |
| Fitted annual increase in incidence (cases per 100,000 births) | 0·32 | 0·16 | 0·20 |
| $\chi^2$ (1 d.f.) for linear increase compared with constant incidence | 1·31 | 0·68 | 2·16 |

*P*
* <0·05
*** <0·001

FIG. 1.—Estimated annual incidence rates for lymphoid leukaemia in boys by 5-year age group, for years of birth for which data are available for all 5-years of the age group. Linear trends are fitted using all the data of Table III (i).

There are few comparable data available from other sources that can be used to verify or refute these findings.

Birch *et al.* (1981) analysed data from the North West Region of England, using data from the Manchester Children's Tumour Registry, which is well known for its completeness of ascertainment and diagnostic accuracy. These authors reported an increase between the years

FIG. 2.—Estimated annual incidence rates for lymphoid leukaemia in girls by 5-year age group, for years of a birth for which data are available for all 5 years of the age group. Linear trends are fitted using all the data of Table III (ii).

1963 and 1977; this was most pronounced among boys aged 1–4 years.* However, their paper does not support the suggestion that there has been a decrease at the upper end of the age range (*i.e.* at 10–14 years).

Clemmesen (1965, 1969, 1974, 1977) has presented registration data for Denmark for the period 1943–1972. This suggests that for boys aged 0–4 years, but not for girls, there was an increase in incidence for children born after about 1962.

Ericsson *et al.* (1978) found no increase in leukaemia incidence between 1958 and 1974 for Swedish children aged 0–14 years. However, their data are consistent with the possibility that, for both boys and girls, incidence rates increased in the age group 0–4 years and decreased at ages 10–14 years.

In summary, it appears that in Great Britain there has recently been an increase in the incidence of lymphoid leukaemia among boys aged 0–4 years, little change for those aged 5–9 and a decrease for those aged 10–14; for girls the changes appear to be much smaller.

It is unlikely that the observed changes are attributable to artefects of registration procedures, since it is hard to see how these could produce an increase in incidence in one group and a decrease in another. In our view the results reported here suggest that there has indeed been a real increase in the incidence of childhood leukaemia for children born from about 1964 onwards, at least for the age group 0–4 years. Data from other sources give some support to the suggestion that such an increase has occurred.

It will be some years before data become available to determine whether

* In the present paper the age range starting at the first birthday and finishing just before the fifth is referred to as 1–4, whereas in Birch *et al.* it is called 1–5.

TABLE V.—*Results of fitting linear and quadratic models to cohort-based incidence rates, allowing for incomplete data from cohorts and assuming that proportional changes in incidence are the same throughout the age range* 0–14 *years*

| | Males age 0–14 | Females age 0–14 |
|---|---|---|
| $\chi^2$ (2 d.f.) for quadratic model | 14·42*** | 4·05 |
| $\chi^2$ (1 d.f.) for quadratic deviation from linear model | 8·40** | 0·28 |
| $\chi^2$ (1 d.f.) for linear model | 6·02* | 3·77 |

*P*
* < 0·05
** < 0·01
*** < 0·001

the increase in incidence at ages 0–4 years, which appeared to start for children born around 1964, extends throughout the 0–14 age range. There is, however, some slight suggestion that the age group 5–9 years is already being affected, but of course few of the children born in the relevant years have yet attained ages over 5 years. In this context it should be noted that the *decrease* in the 10–14 age group, which in our data affects cohorts born between about 1956 and 1966, can also be seen for the two younger age groups (0–4 and 5–9 years) in these cohorts in the *mortality* data presented by Adelstein and White (1976), though the relevant data relate to years when effective therapy was starting to be introduced and mortality rates became affected by improved survival.

There are three possibilities concerning the nature of the recent increase:

(i) that the increase will indeed be observed at higher ages.
(ii) that the increase will be confined to lower ages. This could happen if the increase is due to an aetiological factor which is encountered very early in life or before birth.
(iii) that the increase in the lower age groups is attributable to the earlier occurrence of cases which would otherwise have occurred at a later age; *i.e.* that there is a shift in

FIG. 3.—Lymphoid leukaemia in males aged 0–14 years for cases diagnosed in years 1968–78, AA, Mean age-adjusted annual incidence rate. BB, Estimated linear trend in age-adjusted incidence rate for successive birth years. CC, Estimated quadratic trend in age-adjusted incidence rate for successive birth years.

incidence from older to younger children, perhaps because "susceptible individuals" encounter at an earlier age some, possibly new, leukaemogen in the environment.

It is at present impossible to distinguish between these hypotheses; indeed it may never be possible to distinguish between (i) and (iii), since if the explanation is indeed that the time of diagnosis is "anticipated" in the way suggested in (iii) across the whole of the age range, this could appear as a general increase in incidence.

All these hypotheses assume the occurrence of a leukaemogen which has been recently introduced into, or become more common in, the environment, affecting very young children or pregnant mothers, or possibly with a pre-conception effect. During a period when mortality from infectious diseases has been low, it is implausible that the increase could be due simply to more cases being recognized or to improved diagnosis. There is no evidence that radiation could be a major cause at a time when the rate of prenatal irradiation has been considerably lower than that prevailing during the 1950s. Although various other environmental factors have been suggested as causes of childhood leukaemia, no cause is known for the greeat majority of cases, and there is no obvious explanation for the findings reported here.

Increases in acute myeloid leukaemia among adults in the early 1970s have been reported for England and Wales (Leck *et al.*, 1980), Scotland (Kemp *et al.*, 1980) and for all acute leukaemias taken together, Sweden (Brandt *et al.*, 1979). This increase has not been found in Denmark (Clemmesen, 1979).

In view of the current paucity of information about aetiological factors in both childhood acute lymphoid leukaemia and adult acute myeloid leukaemia, we can offer no useful comment on possible relationships between the trends found in these two diseases. Nevertheless, epidemiological studies of either disease should consider the possibility that these findings are related.

Epidemiological investigations of childhood cancer are in progress, including a case–control study of newly diagnosed patients in 3 Health Service regions. It is possible that these will indicate factors that might be responsible for the increased incidence of childhood acute lymphoid leukaemia. When cancer registration data become available for further years we shall repeat the analysis of leukaemia incidence, in order to determine whether the increase reported here is continuing, and whether there have been similar trends in the incidence of leukaemia in older children.

We thank the Office of Population Censuses and Surveys (Cancer Registration Department), the Information Services Division of the Common Services Agency of the Scottish Health Service, and the many regional cancer registries, consultants and general practitioners who provided the information on which this paper is based. We are grateful to Dr L. M. Kinnier Wilson for providing data from the Oxford Survey of Childhood Cancer, to Mrs E. M. Roberts for her part in collecting the medical records and for secretarial help, and to Mrs B. Dwyer for secretarial help.

The Childhood Cancer Research Group is supported by the Department of Health and Social Security and the Scottish Home and Health Department. Collection of data was also supported by the Marie Curie Memorial Foundation.

### REFERENCES

Adelstein, A. & White, G. (1976) Leukaemia 1911–1973: Cohort analysis. *Population Trends*, **3**, 9.

Birch, J. M., Swindell, R., Marsden, H. B. & Morris Jones, P. H. (1981) Childhood leukaemia in North West England 1954–1977: Epidemiology, incidence and survival. *Br. J. Cancer*, **43**, 324.

Brandt, L., Nilsson, P. G. & Mitelman, F. (1979) Trends in incidence of acute leukaemia. *Lancet*, ii, 1069.

Clemmesen, J. (1965, 1969, 1974, & 1977) Statistical studies in malignant neoplasms *Acta Pathol. Microbiol. Scand.*, Suppl **174**, I & II; Suppl. **209**, III; Suppl. **247**, IV; Suppl. **261**, V.

Clemmesen, J. (1979) Cluster of myeloid leukaemia. *Lancet*, ii, 1238.

Ericsson, J. L-E., Karnström, L. & Mattsson, B. (1978) Childhood cancer in Sweden, 1958–74: I Incidence and mortality. *Acta Paediat. Scand.*, **67**, 425.

Fernbach, D. J. (1977) Natural history of acute leukaemia. *In Clinical Pediatric Oncology* 2nd Edn, (Eds. Sutow *et al*). St Louis: Mosby. p. 300.

FREEDMAN, L., DENT, N. A., HUNT, C., PAYNE, P. M. & SMITH, P. G. (1974) Incidence of childhood leukaemia. *Lancet*, i, 1059.

HEWITT, D. (1955) Some features of leukaemia mortality. *Br. J. Prev. Soc. Med.*, **9**, 81.

KEMP, I. W., STEIN, G. J. & HEASEMAN, M. A. (1980) Myeloid leukaemia in Scotland. *Lancet*, ii, 732.

LECK, I., MORRIS JONES, P. H., STEWARD, J. K., EVANS, D. I. K. & MARSDEN, H. B. (1973) Childhood leukaemia in Manchester. *Lancet*, ii, 509.

LECK, I., BENN, R. T. & SMITH, A. (1980) Changes in myeloid leukaemia incidence? *Lancet*, ii, 749.

MARSDEN, H. B. & STEWARD, J. K. (1976) *Tumours in Children*, 2nd Edn. Berlin: Springer-Verlag, p. 60.

STEWART, A. M. (1961) Aetiology of childhood malignancies: Congenitally determined leukaemias *Br. Med. J.*, i, 452.

STILLER, C. A. & DRAPER, G. J. (1980) Variations in the incidence of childhood leukaemia. *J. Epidemiol. Commun. Health*, **34**, 152.

WATERHOUSE, J. A. H. & POWELL, J. (1973) Incidence of childhood leukaemia. *Lancet*, ii, 1274.